# Hemocompatibility of β-Cyclodextrin-Modified (Methacryloyloxy)ethyl Phosphorylcholine Coated Magnetic Nanoparticles

**DOI:** 10.3390/biom13081165

**Published:** 2023-07-25

**Authors:** Shuhui Li, Mehdi Ghaffari Sharaf, Elyn M. Rowe, Katherine Serrano, Dana V. Devine, Larry D. Unsworth

**Affiliations:** 1 Department of Chemical and Materials Engineering, University of Alberta, Edmonton, AB T6G 1H9, Canada; shuhui1@ualberta.ca (S.L.);; 2 Department of Pathology and Laboratory Medicine, University of British Columbia, Vancouver, BC V6T 1Z7, Canadakatherine.serrano@blood.ca (K.S.); ddevine@pathology.ubc.ca (D.V.D.)

**Keywords:** polymer-coated magnetic nanoparticles, cyclodextrin, protein adsorption, uremic toxin

## Abstract

Adsorbing toxins from the blood to augment membrane-based hemodialysis is an active area of research. Films composed of β-cyclodextrin-co-(methacryloyloxy)ethyl phosphorylcholine (p(PMβCD-co-MPC)) with various monomer ratios were formed on magnetic nanoparticles and characterized. Surface chemistry effects on protein denaturation were evaluated and indicated that unmodified magnetic nanoparticles greatly perturbed the structure of proteins compared to coated particles. Plasma clotting assays were conducted to investigate the stability of plasma in the presence of particles, where a 2:2 monomer ratio yielded the best results for a given total surface area of particles. Total protein adsorption results revealed that modified surfaces exhibited reduced protein adsorption compared to bare particles, and pure MPC showed the lowest adsorption. Immunoblot results showed that fibrinogen, α1-antitrypsin, vitronectin, prekallikrein, antithrombin, albumin, and C3 correlated with film composition. Hemocompatibility testing with whole blood illustrated that the 1:3 ratio of CD to MPC had a negative impact on platelets, as evidenced by the increased activation, reduced response to an agonist, and reduced platelet count. Other formulations had statistically significant effects on platelet activation, but no formulation yielded apparent adverse effects on hemostasis. For the first time, p(PMβCD-co-MPC)-coated MNP were synthesized and their general hemocompatibility assessed.

## 1. Introduction

**Figure 1:**
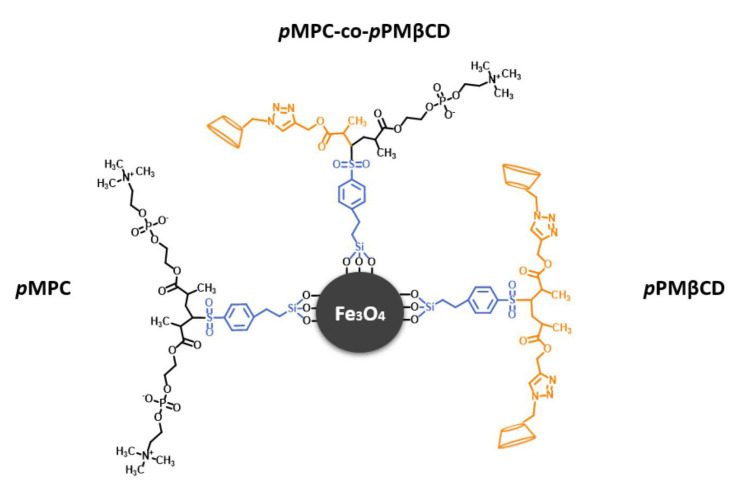
Illustration of MNPs grafted with poly(2-(methacryloyloxy)ethyl phosphorylcholine) and poly(β-cyclodextrin).

Hemodialysis is a costly and time-consuming therapy that ultimately cannot clear all accumulated molecules from the blood of patients with kidney dysfunction [1]. The buildup of these compounds (i.e., uremic toxins [2]) exacerbates kidney dysfunction and directly leads to the progression of chronic kidney disease [3]. Adsorbent surfaces are designed to clear these compounds from the blood, which may transform both treatment costs and patient outcomes associated with hemodialysis [4]. While 2-(methacryloyloxy)ethyl phosphorylcholine (MPC) is a gold-standard low-fouling film [5,6], it is not optimized for uremic toxin clearance. Herein, we synthesized copolymers of β-cyclodextrin (CD) and MPC on magnetic nanoparticles (MNPs) (Figure 1) for the express purpose of studying the effect that CD incorporation has on the hemocompatibility of the particles as an initial screen to determine their potential utility in the context of treating patients with kidney failure. The MNPs provide a platform for removing adsorbed toxins from the blood. General hemocompatibility of these engineered surfaces were assessed through investigating protein–surface interactions and the response of cellular components of the blood.

Nonspecific protein adsorption at the interface of blood-contacting biomaterials can lead to undesired host responses and functional failure of the device [7,8]. For example, plasma protein adsorption on engineered surfaces can lead to microenvironmental changes and protein denaturation, making the introduction of anti-fouling properties essential for the prolonged functionality of various biomaterials, including biomedical implants, biosensors, and membranes [9,10,11]. Common surface modifications used to inhibit protein adsorption include hydrophilic polymers like zwitterionic polymers, including poly(sulfobetaine methacrylate) (pSBMA) and pMPC [12]. However, it is unclear how the formation of surface films composed of CDs and pMPC interact with blood components. Although both CD and MPC monomers exhibit properties that are associated with inhibiting protein adsorption, such as abundant hydroxyl groups found on CDs [13], and the properties associated with the zwitterion of MPC [14,15], further examination of their joint contribution to outcomes related to both proteins and blood cells is crucial to their application in capturing uremic toxins. Circular dichroism was used to examine the effect film properties had on the adsorbed protein structure using albumin (HSA), α-lactalbumin, and lysozyme. These three proteins allow for the surface to be interrogated on different size scales, as HSA and α-lactalbumin have similar charges but different sizes. In a complementary manner, through using lysozyme and α-lactalbumin, the film was evaluated for proteins of a similar size but a different charge [16]. Adsorption-induced rearrangement of HSA was also evaluated using the changes in the fluorescence of tryptophan [17].

In addition to single-protein experiments, understanding how plasma proteins are affected by surface chemistry is crucial to their eventual application. Plasma is a highly intricate fluid that contains a plethora of proteins that can, upon interacting with surfaces, trigger a multitude of deleterious host responses [18]. When combined with platelet activation, these surfaces could initiate thrombus formation, leading to embolisms [19,20]. Therefore, a comprehensive understanding of the interaction between biomaterials, plasma proteins, and platelets is crucial for developing safe and effective medical devices. To this end, the adsorbed plasma proteome was assessed *via* incubation of MNPs with platelet-poor human plasma using sodium dodecyl-sulfate polyacrylamide gel electrophoresis (SDS-PAGE) and immunoblot. The BCA protein assay was employed to determine the total amount of plasma protein adsorbed by different types of MNPs. To probe the platelet function and the more general hemocompatibility of the series of coated MNPs, several complementary techniques were used following incubation with whole blood: complete blood counts, whole-blood rotational thromboelastometry (ROTEM), platelet activation and responsiveness to adenosine diphosphate (ADP) through flow cytometry, and hemolysis through spectrophotometry. Taken together, the results from these experiments depict, for the first time, an overall biocompatibility of the different combinations of CD and pMPC on MNPs for their potential use in uremic toxin clearance.

## 2. Materials and Methods

Chemicals for synthesis: *p*-Toluenesulfonyl chloride (TsCl, reagent grade, ≥98%, Sigma Aldrich, St. Louis, MO, USA), 2-(4-chlorosulfonylphenyl) ethyl trichlorosilane (CTCS, 50% in toluene, Gelest, Morrisville, NC, USA), Sodium azide (≥99%, Fisher Scientific, Waltham, MA, USA), propargyl methacrylate (PM, 98%, Alfa Aesar, Tewksbury, MA, USA), β-cyclodextrin (≥97%, Sigma Aldrich), 2,2′-bipyridyl (bpy, >99%, copper(I)bromide (> 98%), copper(II)bromide (99%), Tris[(1-benzyl-1H-1,2,3-triazol-4-yl)methyl]amine (TBTA, Trichemicals, Edmonds, WA, USA), Dialysis Tubing 500 MWCO (Cole-Parmer, Vernon Hills, IL, USA), Lewatit TP 207 adsorbent, TetrakisacetonitrilecopperI hexafluorophosphate (Cu(CH_3_CN)_4_PF_6_, Sigma Aldrich, St. Louis, USA), 2-methacryloyloxyethyl phosphorylcholine (MPC, Sigma Aldrich, St. Louis, MO, USA), ultra-thin carbon-coated copper grid (150 mesh, Ted Pella, Inc., Redding, NC, USA), sodium hydroxide (Fisher Scientific, Waltham, MA, USA), FeCl_2_**ꞏ**4H_2_O, FeCl_3_**ꞏ**6H_2_O, and ammonium hydroxide solution (25%) were purchased from Sigma Aldrich, St. Louis, MO, USA.

Chemicals for experiments: Sodium phosphate dibasic heptahydrate, sodium phosphate monobasic monohydrate, and PBS tablet were purchased from Fisher Scientific. Platelet-poor human plasma was procured via the Blood4Research program from Canadian Blood Services. Sodium dodecyl sulfate (SDS) and polyvinylidene fluoride (PVDF) membrane were from Bio-Rad, Hercules, CA, USA. The TMB-stabilized substrate was from Promega, Madison, WI, USA, and the BCA protein assay from the Pierce™ BCA Protein Assay Kit (Thermo Fisher Scientific Inc., Waltham, MA, USA) was used. For the complete list of antibodies, see Table S1 in the Supplementary Materials. Statistical analysis of protein adsorption data was performed using one-way ANOVA, followed by a post-hoc Tukey’s HSD test, with a significance level of *p* < 0.05.

### 2.1. Polymer Synthesis and Fixation

PMβCD monomer and bare MNPs were prepared following previously reported methods [21,22,23]. Bare MNPs were decorated with CTCS: 500 mg of bare MNPs were placed in an argon-filled three-neck flask, and 5 mL of CTCS solution was added drop-wise and left to react for 3 h. MNP-CTC was washed thrice with THF and twice with ethanol through consecutive separation and redispersion. The MNP-CTC product was dried in the vacuum overnight and sealed under an argon blanket for storage. Atom transfer radical polymerization (ATRP) synthesis methods were applied for polymer synthesis, as presented in Table 1. Before the experiment, argon was added to a round-bottom flask thrice to remove any gas present and ensure an argon blanket. MPC and PMβCD were then dissolved in a 10 mL mixed solvent, which contained an even volume of water and ethanol. The solution was stirred and bubbled with argon for 20 min to form the MPC-PMβCD complex.

### 2.2. Polymer Characterization

TEM (JEM-ARM200CF S/TEM, JEOL, Houston, TX, USA) analysis used an accelerating voltage of 200 kV to obtain images of particles. These images were further analyzed using ImageJ. To prepare the samples, a droplet of a well-dispersed sample was deposited onto an ultra-thin carbon-coated copper grid and allowed to air-dry for 24 h prior to characterization.

The zeta potential of the engineered surfaces was evaluated using the Malvern Zetasizer Nano-ZS (Nano ZS, Malvern Instruments, Malvern, UK). To prepare the sample, 25 μL of a 1 mg/mL MNPs suspension was mixed with 3 mL of DI water and subjected to 30 s of ultrasonication before the zeta potential measurement. Three repeats were conducted, with each measurement comprising 12 runs each. These were averaged to obtain the reported zeta potential.

### 2.3. Circular Dichroism

Far-UV CD (DSM 17 Circular Dichroism spectrometer, Olis, Athens, GA, USA) spectra of HSA, α-lactalbumin, and lysosome with the polymer-coated MNPs were obtained using human serum albumin: 1.25 mg/mL, and a-lactalbumin and lysozyme: 0.25 mg/mL. The MNP concentration was 0.25 mg/mL. The same volumes of protein solution and MNP solution were incubated for 3 h at 37 °C before the test. The spectra were recorded from 180 to 260 nm. The presented results are an average value of three independent repeats. CDNN 2.0 software was used to determine the secondary structure changes upon adding MNPs.

### 2.4. Fluorescence Spectroscopy

The binding interaction study of nanoparticles synthesized with HSA was conducted in vitro using a previously described procedure [24]. Briefly, a solution of HSA (330 µg/mL in 10 mM of PB, pH 7.4) was titrated with different formulations of nanoparticles (1 mg/mL). Fluorescence spectroscopy (FlexStation 3 multimode plate reader) was used to study the interaction between the protein and the nanoparticles. The reduction of fluorescence intensity was recorded at emission scanning wavelengths from 300 to 500 nm, λex = 295 nm. The binding constant (K_b_) and the number of binding sites (n) were determined according to previously published methods (Equation (1)) [25,26,27]:

(F_0_ − F)/(F_0_ − F_s_) = [(S)/K_d_]n (1)

where F_0_ is the relative fluorescence intensity (F) of the protein solution alone, F_s_ is the relative fluorescence intensity of protein saturated with MNPs, and [S] is the concentration of MNPs. n is the number of binding sites and was determined from the slope of the plot, log [(F_0_ − F)/(F − F_s_)] vs. log [S]. The log [S] at log [(F_0_ − F)/(F − F_s_)] = 0 determines the logarithm of the dissociation constant (K_d_), where K_b_ is the reciprocal of K_d_.

### 2.5. SDS-PAGE and Immunoblot

Upon pooled platelet-poor plasma arrival, plasma samples were aliquoted and stored at −80 °C until use. The study was approved and conducted per the guidelines set by the research ethics board of the University of Alberta. Nanoparticle and platelet-poor plasma incubation was carried out according to existing protocols [28]. Briefly, different magnetic particles were added to 37 °C plasma at the same concentration used for the recalcification turbidimetric assay (0.18 mg/mL) and incubated at 37 °C for 2 h. Samples were subsequently centrifuged at 20,000× *g* for 10 min. The supernatant was removed, and nanoparticles were washed twice with 1 mL of PBS to remove loosely bound proteins. The final pellets of particles and adsorbed proteins were resuspended in 100 μL of 10% SDS in PBS and incubated at 50 °C for 2 h to elute the adsorbed proteins from the surface of the nanoparticles. The eluted protein sample concentration was quantified using the Pierce™ BCA protein (detergent-compatible) assay. The final sample was further analyzed using SDS-PAGE and immunoblotting.

The analysis of samples using SDS-PAGE and immunoblot techniques was conducted using a protocol described previously [28,29]. Before SDS-PAGE, a denaturing sample buffer containing SDS and 0.5 M β-mercaptoethanol was added to each sample and heated at 95 °C for 5 min. A constant amount of each protein sample (30 μg) was run on 12% polyacrylamide gels. Samples were transferred onto 0.2 μm-pore size immunoblot polyvinylidene difluoride membranes (Bio-Rad Laboratories, Inc., Hercules, CA, USA). Upon electro-transfer, each membrane was divided into 23 strips, with 2 used for colloidal gold staining and the rest for immunoblot, using 21 individual proteins (Table S1, Supplementary Material). Each primary antibody was used at a 1:1000 dilution. Horseradish peroxidase (HRP)-conjugated secondary antibodies and 3,3′,5,5′-tetramethylbenzidine (TMB)-stabilized chromogen substrate (Promega) were used for the visualization of the immunoblot results. The color development time was kept consistent between immunoblots to compare the results between different samples. The strips were dried, assembled, and digitized immediately after stopping the color development with water.

### 2.6. Fibrin Clot Formation in Plasma

Clot formation was assessed using the plasma calcification turbidimetric assay. Plasma (100 μL) was incubated with MNPs. Plasma was incubated with PBS (10 mM) for 30 min, then incubated with MNPs for 1 h prior to testing. Then, 100 μL of 0.025 M CaCl_2_ was injected into a 96-well plate for the turbidity reading. A BioTek ELx808 plate reader was used to measure the absorbance at 405 nm at 1 min intervals over 1 h. All steps were performed at 37 °C, with three independent repeats.

### 2.7. Whole-Blood Hemocompatibility Testing

Venous blood from three healthy donors was collected into 2.7 mL BD vacutainers containing buffered sodium citrate (0.109 M, 3.2%) for hemocompatibility evaluation. This study was approved by the UBC Clinical Research Ethics Board (H22-00215), and all donors provided informed consent. For whole-blood hemocompatibility testing, MNPs dispersed in water (or an equal volume of deionized water, as a control) were added to 2.7 mL of citrated whole blood at a final concentration of 0.18 mg/mL and incubated on a rocker for 1 h at 37 °C.

Following incubation, complete blood counts were obtained using a Sysmex XN-550 hematology analyzer (Sysmex Corporation). Coagulation was assessed in the treated citrated whole blood using rotational thromboelastometry (ROTEM; Instrumentation Laboratory, Bedford, MA, USA), where 300 μL of whole blood was mixed with the STAR-TEM and EXTEM reagents to re-calcify and activate the extrinsic coagulation pathway, respectively. Primary readouts included: the clotting time (the period from the beginning of coagulation to the start of fibrin polymerization), the clot formation time (velocity of clot formation—platelet-dependent), and the maximum clot firmness (mechanical strength of the clot—dependent on platelet function fibrin polymerization, and Factor XIII activity).

Platelet activation and responsiveness were evaluated using flow cytometry on a BD FACSCanto II flow cytometer (BD Biosciences). For baseline activation, 3 μL of whole blood was incubated with 5 μL of mouse anti-CD62P (IM1759U, Beckman Coulter, Brea, CA, USA) in 0.22 μm-filtered PBS at a total volume of 50 μL. For responsiveness, 3 μL of whole blood was incubated with ADP (Chrono-Log, Havertown, PA, USA) at a final concentration of 10 μM and 5 μL of antibody in 0.22 μm-filtered PBS, at a total volume of 50 μL. After a 30-min incubation at room temperature, samples were diluted with 1 mL of 0.22 μm-filtered PBS before measurement. The platelet population was confirmed with a CD41 stain and gated for the remainder of the experiments. Gates for a positive CD62P signal in the platelet population were determined for each experiment based on an isotype control (IgG1; IM0670U, Beckman Coulter).

The remaining whole blood was centrifuged at 3000 rpm for 10 min at 4 °C to obtain platelet-poor plasma (PPP), then the PPP was spun again at 20,000× *g* for 20 min at 4 °C to pellet the remaining microparticles before exposure to a magnet for 15 min at room temperature. Hemolysis was assessed in PPP using the previously described Harboe method [30], and C3a levels reflecting complement activation were assessed in plasma aliquots frozen at −70 °C using a commercial ELISA (#A031, Quidel, San Diego, CA, USA), following the kit instructions for plasma. All diluted samples were within the detection limits of the ELISA.

Graphing and statistical analysis for the hemocompatibility outcomes were performed in RStudio using R version 4.0.5, with the packages rstatix, dplyr, grid, and ggplot2. Due to the innate variability across donors, hemocompatibility results were evaluated using a repeated-measures ANOVA, with the donor as the identifier and treatment (MNP) as the within-subject variable. Post hoc pairwise comparisons were performed using paired *t*-tests, where each MNP formulation was compared to the water control. *p*-values were not adjusted for multiple comparisons in post hoc testing, as the goal was to identify any MNP formulations that showed indications of hemo-incompatibility with high sensitivity.

## 3. Results and Discussion

### 3.1. Properties and Characterization of the Modified MNPs

**Figure 2:**
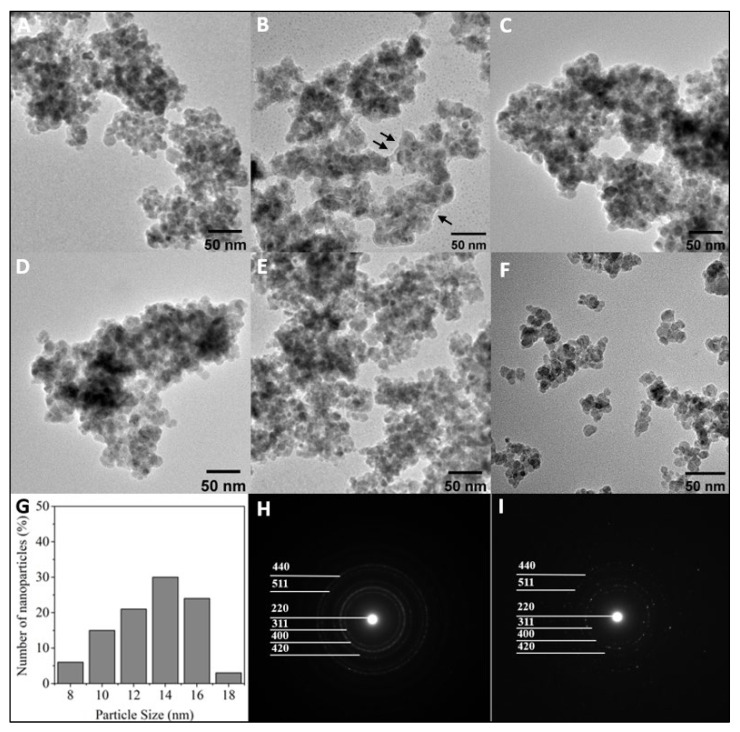
Representative TEM and SAED characterization of bare and polymer-coated MNPs. (**A**–**E**) TEM images of synthesized polymer-modified nanoparticles of samples (**A**–**E**), where the black arrow indicates the polymer layer. (**F**) TEM image of bare MNP. (**G**) Particle size distribution of bare MNP in (**F**). (**H**) SAED pattern of particle E. (**I**) SAED pattern of MNP.

**Figure 3:**
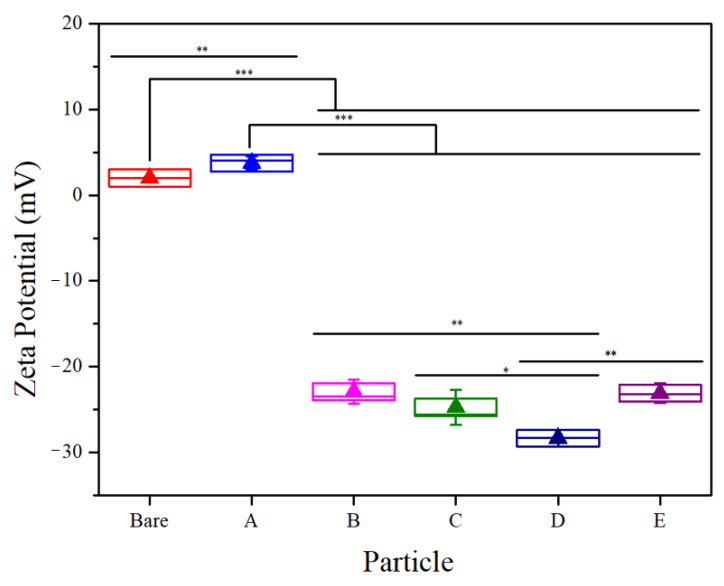
Representative zeta potential test of bare MNP and particles A to E. Unpaired *t*-tests were conducted to compare results across each pair of groups. Comparisons that are not displayed were not statistically significant (* *p* < 0.05, ** *p* < 0.01, *** *p* < 0.001, data presented as mean ± 1 SD, *n* = 3).

A series of polymer-modified particles were synthesized using ATRP techniques. Figure 2 illustrates the representative TEM results for the modified MNPs, where an average particle size of 13.45 ± 2.52 nm was found through analyzing a random subset of particles (*n* = 33). The selected-area electron diffraction (SAED) pattern indicated that both the bare and polymer-coated MNPs had a ring pattern consistent with a polycrystalline structure of magnetite, matching the (220), (311), (400), (420), (511), and (440) planes, as specified by the JCPDS Card No. 19-0629 [31,32]. All particles’ engineered surfaces exhibited a negative net charge, as demonstrated by their zeta potential data (Figure 3). The bare MNP’s zeta potential was significantly different (*p* < 0.05) than all modified surfaces. No significant difference in the zeta potentials of surfaces B, C, and E was observed. The zeta potential of the surface on particle D was significantly (*p* < 0.05) more negative but still similar to other CD:MPC surfaces. Surfaces B–E had a sufficient surface charge to inhibit rapid flocculation in buffers, compared to the control and A surfaces [33,34].

### 3.2. Circular Dichroism

**Figure 4:**
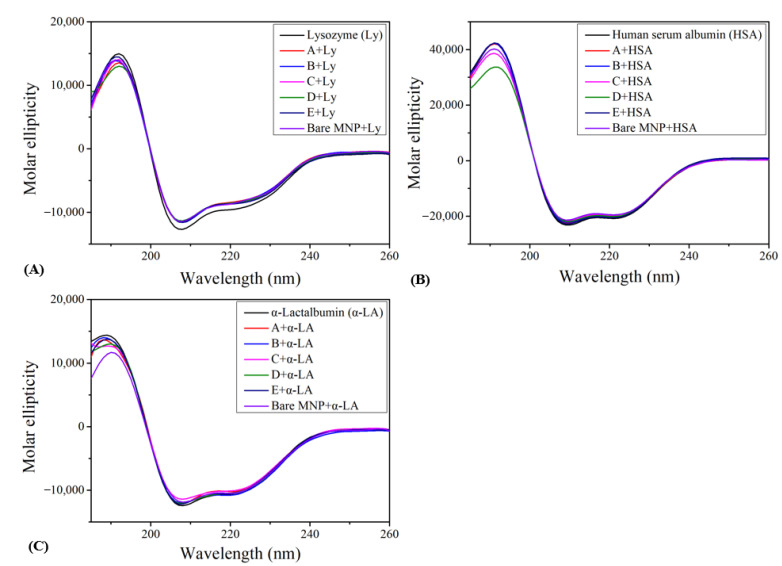
Circular dichroism spectrum of (**A**) lysozyme, (**B**) HSA, and (**C**) α-lactalbumin in the presence of particles A to E and bare MNP (*n* = 3).

The effect of surface chemistry differences between particles A to E and the bare MNP control on the lysozyme, HSA, and α-lactalbumin structure were evaluated (Figure 4, Table 2). Overall, modified MNPs exhibited negligible effects on the secondary structure of all three proteins. In the case of lysozyme, peak absorbance occurred at 192 nm, and a reduction of 2.4~3.4% in the helix and an increase of 1.7~2.4% in the beta-sheet were observed compared to the native protein reference. Concerning HSA, particle D induced the largest reduction in the helix (8.4%), followed by C (3.6%) and E (2.3%). The random coil structure for HSA with particle D was notably increased, by 5.7%. α-Lactalbumin had a peak at 188 nm and minima around 218~223 nm. For α-lactalbumin, the conformational change induced by the engineered surface was less than 1% compared to the native control, except for a 1.7% decrease in the helix with particle D. Bare MNPs induced the most conformational changes in α-lactalbumin, reducing the helix by 3.5%. Among these three proteins, the net-negative-charged HAS structure was affected the most. As a carrier of many substances in the blood, HSA can bind to substances of varied characteristics and have its structure perturbed through this interaction. In contrast, lysozyme and α-lactalbumin remained relatively stable, perhaps due to the smaller sizes (HSA: 66.5 kDa, lysozyme and α-lactalbumin: 14 kDa) and charge densities (HSA: −19, lysozyme (+8), and α-lactalbumin (−7)) relative to HSA [23,35,36,37].

### 3.3. Changes in Intrinsic Fluorescence of HSA upon Adsorption

**Figure 5:**
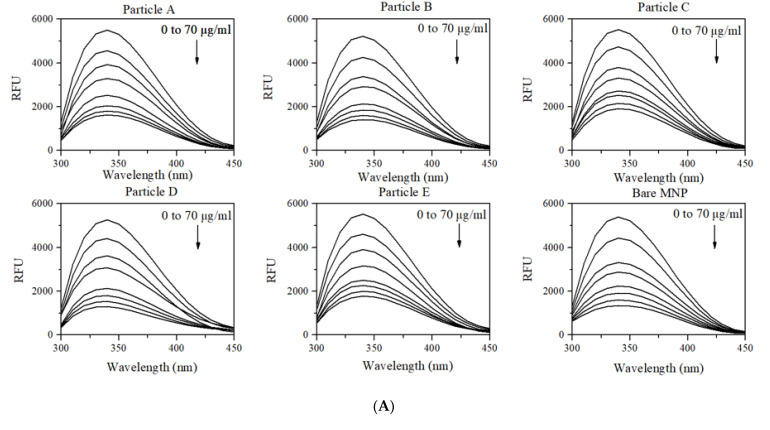

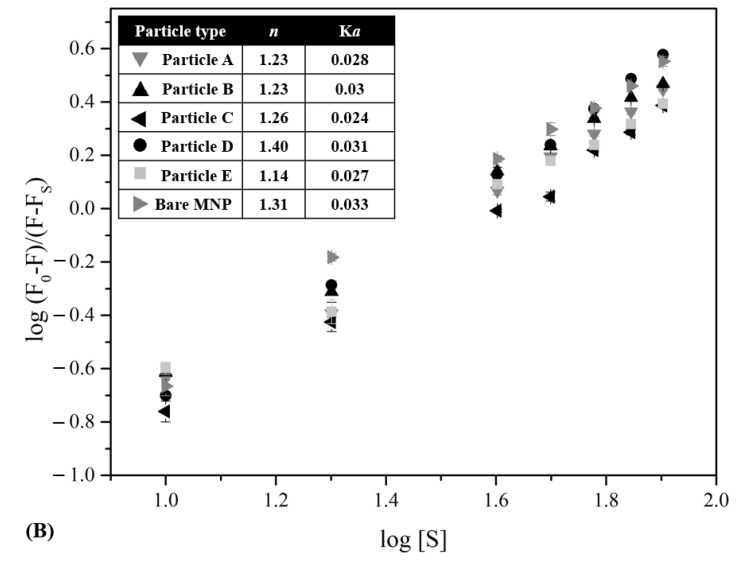
Study of the interaction of HSA protein with MNPs via quenching of the intrinsic fluorescence of HSA in the presence of different concentrations of MNPs. (**A**) Fluorescence emission spectra of HSA solution titrated against increasing concentrations of MNPs in the solution (0–70 μg/mL). (**B**) Binding constant (K_a_) and number of binding sites (n) obtained from the plot, log [(F_0_ − F)/(F − F_s_)] vs. log [S]. RFU = relative fluorescence units, [S] = MNP concentration, F_0_ = relative fluorescence intensity (F) of protein solution alone, and F_s_ = relative fluorescence intensity of protein saturated with MNPs. Data represent mean ± 1 SD, *n* ≥ 3.

Previous studies have shown that the intrinsic fluorescence intensity of tryptophan located in HSA can be reduced with increased adsorption to MNPs, and that this is potentially dependent upon the surface chemistry of particles [25,38]. In this fluorescence study, the quenching effect (Figure 5A) for HSA upon incubation with engineered MNPs and bare MNPs allowed for the determination of the number of binding sites on nanoparticles (n) and the binding constant (K_a_) for each type of MNP (Figure 5B). Particles A and B showed the same number of binding sites, close to the number of binding sites on particle C (ranging from 1.23 to 1.26). Among different types of MNP formulations, particles E and D had the lowest and highest number of binding sites, respectively. Furthermore, we found that particle C had the weakest binding affinity towards HSA, whereas bare MNPs without surface modification exhibited the strongest binding affinity towards HSA (Figure 5B).

### 3.4. Total Adsorbed Protein

**Figure 6:**
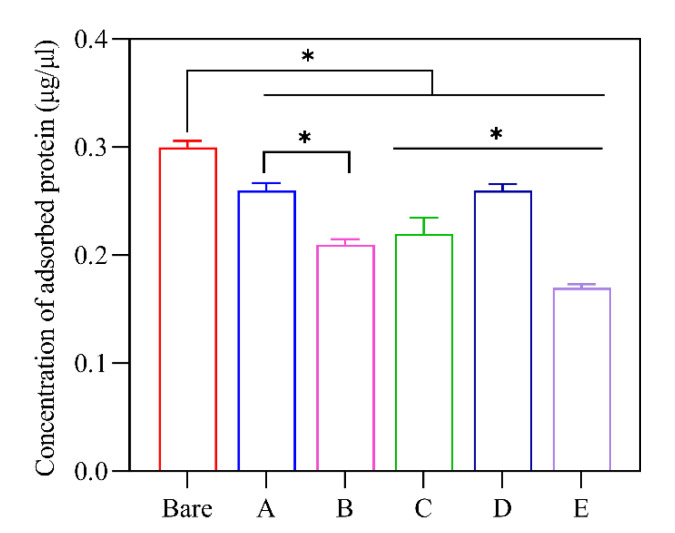
Representative results showing the amount of adsorbed protein determined using the BCA assay. * Represents *p* < 0.05, data represent mean ± 1 SD, *n* ≥ 3.

A detergent-compatible BCA assay was used to quantify the concentration of eluted plasma proteins from each type of MNP (Figure 6), which ranged from 0.17 to 0.3 mg/mL for a constant surface area of MNP. Bare MNP showed the highest, and particle E showed the lowest values of eluted proteins. A significant reduction (*p* < 0.05) in protein adsorption occurred for all modified nanoparticles compared to the unmodified bare nanoparticles. Although particle size has been shown to influence protein binding [39], the particle size and surface area of these MNP systems were relatively similar, and surface chemistry likely dominated the adsorption of proteins. Notably, the protein adsorption of MNPs displayed an inverse relationship with the content of MPC, with bare particles exhibiting the highest plasma protein adsorption and particle E showing the lowest. Intermediate MPC-containing films displayed a gradual decline in the total adsorbed amount of protein, except for particle D. Nonetheless, there was a significant difference between the protein content adsorbed by bare particles and particle D. These findings correlate with previously reported results, where MPC-only modified surfaces have shown significantly improved inhibition of protein adsorption [40,41].

### 3.5. Plasma Clotting in the Presence of Polymer-Coated Nanoparticles

**Figure 7:**
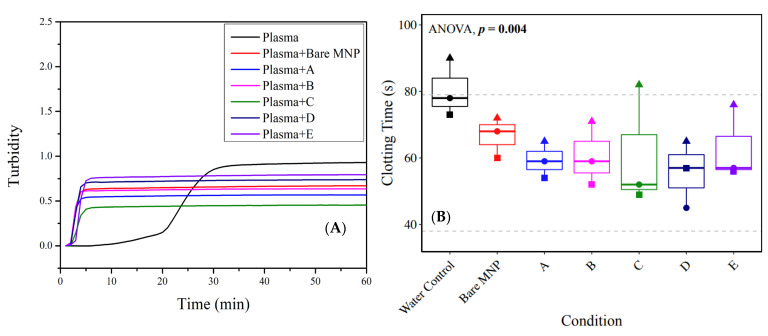
The presence of MNPs reduced the clotting time, driven by plasma proteins. (**A**) Representative plots of baseline-corrected average clot formation in platelet-poor human plasma over 60 min, with particles A to E or bare MNP present. (**B**) Whole-blood ROTEM results for clotting time—reflective of fibrinogen polymerization. Shapes reflect biological replicates. Results were compared across groups with repeated-measures ANOVA to compare differences within biological replicates across groups, and paired *t*-tests were used for pairwise comparisons to the water control. Comparisons not shown were not statistically significant.

Intrinsic contact activation through the interaction of the surface, Factor XII, high-molecular-weight kininogen, and prekallikrein is a known humoral response to artificial materials that leads to thrombus formation [42,43]. Plasma clotting experiments were conducted for bare and engineered MNPs (Figure 7, Table 3). Considering the obvious surface area effect on clot kinetics, clot formation upon MNP incubation was much quicker than that of the plasma control, but surprisingly led to a reduced clot intensity. Native plasma showed that clotting initiated at 10 min and reached a plateau at 30 min, with a turbidity of 0.93. Overall, adding MNPs advanced the clotting time but inhibited the final clot turbidity. Internal comparisons of the polymer film on systems with similar surface areas showed that particles A, D, and E showed a clotting start time at 2 min, whereas particles B and C showed clotting at 1 min. The plateaus were reached at 5 min for particles A, B, and C, and 6 min for D, E, and bare MNP. From the turbidity results, it was observed that particle D inhibited clot formation the most compared to the other particles. Particle E exhibited the best compatibility, as demonstrated by the lowest clotting inhibition effect. This was predictable as it was coated with solely the MPC polymer, which was introduced to improve the overall anti-fouling and biocompatibility. In the co-polymer groups, particle C showed the highest clotting inhibition effect. Coagulation was also assessed in whole blood through rotational thromboelastometry (ROTEM), where a similar effect was observed—the addition of any type of MNP decreased the time to fibrinogen polymerization (clotting time) compared to the control (whole blood with an equivalent volume of water added) (Figure 7B).

### 3.6. Quantification of Protein Adsorption

The immunoblot band intensity data for all MNPs were quantified using a 13-step gray-scale system, where zero indicates no visible band, and 12 indicates the highest band intensity. To facilitate the band intensity comparison between different systems, the amount of loaded protein and the color development time were kept constant for all systems studied (Table 4).

Albumin (66.5 kDa) is the most prevalent plasma protein (35–50 g/L) and is largely responsible for binding various metabolic compounds, lipids, and even medications [44]. Moreover, albumin is known to adsorb to surfaces, which alters the subsequent binding of other proteins and even affects coagulation [45,46]. For example, albumin adsorption to polyacrylonitrile hemodialysis membranes has reduced the adhesion and aggregation of platelets to the membrane [47]. Previous research using immunoblotting techniques has shown that albumin has a high affinity to elastin-like polypeptide nanoparticles, requiring the dilution of primary and secondary antibodies [28]. Herein, albumin adsorption was not sufficient enough to require a dilution of antibodies for characterization. Studies conducted on siliconized glass and polycarbonate membrane surfaces have shown that albumin has a higher affinity for hydrophobic surfaces [48,49]. Our results surprisingly showed that as the MPC content in the film increased, the adsorbed albumin increased to levels similar to the control bare MNP surface. This is contrary to studies that have shown that the addition of β-cyclodextrin to hydroxyapatite nanoparticles enhanced albumin adsorption [50].

#### 3.6.1. Immune Response-Related Proteins

**Figure 8:**
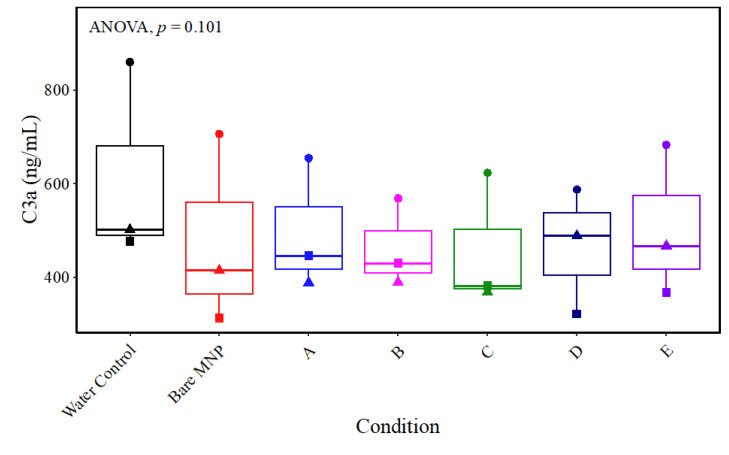
C3a ELISA results of MNP-depleted plasma from whole-blood hemocompatibility studies. Whole blood from *n* = 3 healthy donors was incubated with 0.18 mg/mL of each MNP formulation, and MNP-depleted plasma was assayed for C3a. Results were compared across groups with repeated-measures ANOVA to compare differences within biological replicates across groups, and paired *t*-tests were used for pairwise comparisons to the water control. Comparisons not shown were not statistically significant.

The activation of the complement system is accomplished through the concerted action of multiple proteins, which operate through three distinct activation pathways: the classical pathway, the alternative pathway, and the mannose-binding lectin pathway [51]. These cascades mount a defense against bacterial infection and the clearance of immune complexes and apoptotic cells. It also links innate and adaptive immune responses as complement component 3 (C3) plays a key role in all three activation pathways, particularly in the alternative pathway, which is involved in biomaterial-induced complement activation [28,52]. Prolonged blood exposure to synthetic surfaces during hemodialysis can lead to chronic inflammation due to chronic activation of the complement system [53]. Four distinct bands can be seen for C3: whole C3, 187 kDa, ɑ chain, 115 kDa, β chain, 70 kDa, and an activation fragment, 42 kDa [28]. Herein, intact C3 was not observed for any MNP system. All other bands were present and abruptly decreased in intensity for particles D and E. Bare MNP controls were similar in band intensity to the CD-only film (particle A). The activation fragment (42 kDa) was present for all MNP types and showed a drastic decrease for particles D and E. This suggests that particle D and E MNPs induced less activation of C3 than other surfaces. C3a was also independently assayed via ELISA in three biological replicates of MNP-depleted plasma from whole-blood hemocompatibility studies (Figure 8). One replicate mirrored the results of decreased C3a in bare MNP and particles D and E, but these results were inconsistent in the other two replicates. Taken together with the protein adsorption results from the immunoblot analysis, the independent C3a ELISA results may indicate that while there was an increased presence of C3a with the increased PMβCD:MPC ratio in the MNP coating, there may also be donor-dependent increases in C3a generation with these formulations, considering that the ELISA results on MNP-depleted plasma were not significantly different. However, consistent with the blotting results, a trend toward decreased C3a in the MNP-treated plasma compared to the untreated control was observed, indicating adsorption to all MNP surfaces.

Of the five distinct isotypes of serum immunoglobulins (IgM, IgD, IgG, IgA, and IgE), IgG is the most prevalent in human serum: ~10–20% of total plasma protein [54]. IgG presence leads to the activation of the classical pathway of complement [28]. The light chain of IgG (27 kDa) again showed a step decrease in intensity for particles D and E compared to all other systems. The heavy chain of IgG (55 kDa) was relatively low for all MNP systems, with no trend associated with the MPC content of the surface film. This result is consistent with the role of the content of MPC in inhibiting protein adsorption.

Transferrin (77 kDa) carries iron (ferric iron) throughout the body, is a component of the innate immune system, can activate macrophages, and acts to restrict the survival of bacteria [28,55,56,57]. All types of MNPs showed relatively high and consistent transferrin adsorption, suggesting that this protein’s binding may trigger macrophage activation.

Vitronectin is a multifunctional glycoprotein and, in plasma, acts as a complement regulatory component. Different types of MNPs, including bare particles, showed relatively high-intensity values associated with vitronectin, compared to the low levels observed for particles D and E. Vitronectin is known as a major plasma protein in association with polymer surfaces. Although fibronectin and vitronectin have similar plasma concentrations, studies conducted on various polystyrene-based surfaces have shown that vitronectin has a greater propensity to bind to surfaces [58]. Although MNPs showed moderate to high values for vitronectin, none of the MNPs were found to adsorb fibronectin.

α_1_-Antitrypsin is the most abundant serine protease inhibitor in human plasma, constituting 95% of the trypsin inhibitory capacity. As a serine protease inhibitor, the primary role of α_1_-antitrypsin is to inhibit the proteolytic activity of serine protease neutrophil elastase. Aside from its primary role as a protease inhibitor, α_1_-antitrypsin has other immunomodulatory functions, including anti-inflammatory properties and regulation of T- and B-lymphocytes [59,60]. α_1_-Antitrypsin has been previously found absorbed in large amounts to elastin-like polypeptide nanoparticles, suggesting its inhibitory role in preventing the nanoparticles from degradation by elastase [28]. This protein was found in high binding levels to bare MNP, and particles A, B, and C, but particles D and E showed moderate adsorption levels. It has been previously shown that α_1_-antitrypsin in the protein corona of Au nanoparticles could act as a cell-binding-promoting factor [61,62,63]. Considering the anti-inflammatory role of α_1_-antitrypsin, this protein may reduce macrophage activity [64].

α_2_-Macroglobulin is another component of the innate immune system, regulating proteases by clearing them from the blood [65,66]. No visible immunoblot was identified for this protease. The lack of α_2_-macroglobulin may suggest that MNP surfaces are not playing an active role in promoting either clotting or fibrinolysis, as it acts as an inhibitor for both processes [29].

#### 3.6.2. Coagulation-Related Proteins

Fibrinogen (340 kDa) has a key role in coagulation and is a substrate for three related enzymes, including Factor XIIIa, thrombin, and plasmin. It is a heterodimer, each half consisting of three polypeptide chains (Aɑ, Bβ, and γ), linked via disulfide bonds. Thrombin enzymatically cleaves Aɑ and Bβ chains from the N-terminal, which ultimately leads to the initiation of clot formation. Factor XIIIa stabilizes the fibrin polymers via cross-linking, thus increasing its resistance to degradation through fibrinolysis [67,68]. Fibrinogen appears as three distinct bands: Aɑ, 68 kDa, Bβ, 56 kDa, and γ, 48 kDa. In addition, cleavage fragments appear as bands at <48 kDa [28]. In all MNP systems, the band intensity for all fibrinogen fragments remained relatively constant but decreased for particles D and E. Particles D and E showed no fibrinogen cleavage fragments (<48 kDa). It has been previously shown that coating of polyethylene and polypropylene surfaces with β-CD decreased the adsorption of fibrinogen, potentially enhancing the blood compatibility of those surfaces [69]. However, in this case, it is apparent that films with higher amounts of MPC showed a lower amount of related fibrinogen.

Prothrombin (72 kDa) is a single-chain glycoprotein that is an inactive precursor for thrombin. The proteolytic conversion of prothrombin to thrombin is induced by Factor Xa in the presence of Factor V, phospholipid, and Ca^2+^ [70]. Low levels of prothrombin were observed for all MNP systems, whereas the amount of antithrombin decreased with the increasing MPC composition of the engineered films. Antithrombin (53 kDa) is a serine protease inhibitor that is an endogenous anticoagulant that complexes with thrombin, and other activated coagulation factors, to inhibit coagulation [71].

The contact pathway of coagulation comprises three serine proteinases, namely coagulation Factors XII and XI, prekallikrein, and the non-enzymatic cofactor high-molecular-weight kininogen [72]. Factors XII and XI have been previously reported in low quantities in protein corona of poly(acrylic acid)-coated TiO_2_ nanoparticles. Herein, low and consistent amounts of Factor XII were found on all MNPs, where bare particles showed the highest intensity. Factor XI was relatively strongly bound to all MNP surfaces, with no decrease observed for particles D and E, similar to previous reports for poly(acrylic acid)-coated Fe_2_O_3_ nanoparticles [73]. Prekallikrein, the precursor of kallikrein, cleaves high-molecular-weight kininogen [74] and may be found at 85 kDa, with consistent levels for all modified MNPs that were much lower than the bare MNP control. The kallikrein band (50 kDa) was relatively higher than prekallikrein (85 kDa) for all MNP types. The particle D and E films again showed a sharp decrease in kallikrein intensity. Previous studies have established that the activation of the contact system results in a decrease in the intensity of prekallikrein immunoblot bands, with the formation of complexes of kallikrein with the C1 inhibitor and α_2_-macroglobulin [75].

Fibronectin is a critical component of the fibrin clot, binding to fibrin via non-covalent interactions and covalent cross-linking, leading to the regulation of platelet function and hemostasis [76]. Protein S regulates coagulation, exhibiting anticoagulant function independent of activated protein C, which directly inhibits intrinsic tenase and prothrombinase complexes [77]. None of the MNPs showed adsorption of fibronectin, protein S, or protein C, suggesting a limited clot formation and fibrinolytic response [28].

Plasminogen (91 kDa) is the inactive precursor of plasmin, the major protease that catalyzes the degradation of fibrin clots. Again, particles D and E showed a sharp decline in adsorbed plasminogen compared to the other MNP types. It is thought that surface adsorption of plasminogen might indicate fibrinolytic activity [78,79].

### 3.7. Whole-Blood Hemocompatibility and Impact on Platelet Function

**Figure 9:**
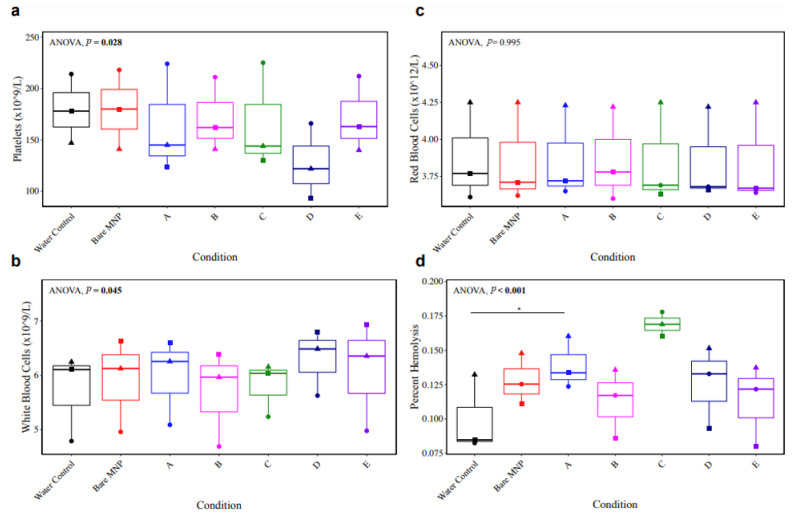
Complete blood counts and hemolysis in response to whole-blood MNP treatment. Whole blood from *n* = 3 healthy donors was incubated with 0.18 mg/mL of each MNP formulation, and complete blood counts were measured on the Sysmex XN-550 hematology analyzer. Results for (**a**) platelets, (**b**) white blood cells, and (**c**) red blood cells are shown. Hemolysis was assayed in MNP-depleted plasma via the Harboe method (**d**). Results were compared across groups with repeated-measures ANOVA to compare differences within biological replicates across groups, and paired *t*-tests were used for pairwise comparisons to the water control (* *p* < 0.05). Comparisons not shown were not statistically significant.

Further characterization was performed in whole blood to assess the hemocompatibility of the MNP formulations based on the published criteria of leukocyte presence, hemolysis, and platelet function—in addition to the results reported above on complement activation and coagulation [80]. Whole blood collected in 2.7 mL of sodium citrate vacutainers from *n* = 3 biological replicates was incubated at 37 °C for 1 h with the same concentration of MNPs as the previous experiments in plasma (0.18 mg/mL), and complete blood counts were performed on the Sysmex XN-550 hematology analyzer. A trend toward a decrease in the platelet number was observed in particle D (Figure 9a), which could reflect platelet aggregation, rendering individual platelets undetectable. No evident trends emerged for differences in leukocyte (Figure 9b) or red cell (Figure 9c) counts, despite a statistically significant repeated-measures ANOVA result for leukocyte counts.

Supernatant hemoglobin levels were measured in MNP-depleted plasma from the MNP-treated whole blood *via* the Harboe spectrophotometric method for quantification of percent hemolysis, based on the total hemoglobin content of the blood measured on the hematology analyzer. Results in Figure 9d demonstrate a trend toward increased hemolysis in MNP-treated whole blood, with a statistically significant increase in the presence of particle A and a larger effect size for particle C, just below statistical significance (*p* = 0.057). Important to note, however, is that the supernatant hemoglobin content for all specimens remained below 0.2 g/L, which is considered to be insignificant hemolysis [81]. Therefore, MNP treatment did not appear to result in a concerning level of hemolysis.

**Figure 10:**
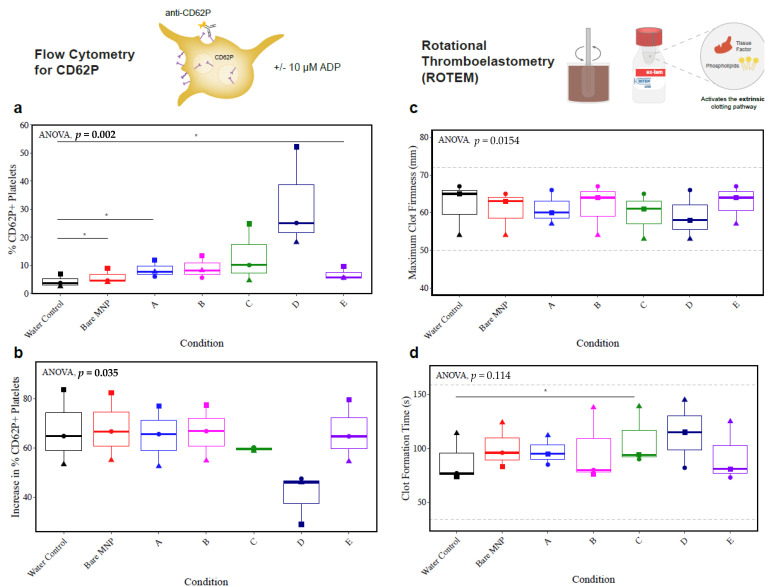
The trend toward increased platelet activation with a lower PMβCD content, without a robust impact on the platelet function in coagulation. Whole blood from *n* = 3 healthy donors was incubated with 0.18 mg/mL of each MNP formulation, then assessed for platelet-related outcomes. (**a**) Baseline platelet activation reflected by the surface expression of CD62P detected by flow cytometry. Percentage of CD62P+ platelets displayed. (**b**) Platelet degranulation in response to 10 μM of ADP, reflected by the surface expression of CD62P, detected by flow cytometry. Baseline activation was subtracted from the % of CD62P+ platelets to yield an increase in the degranulated platelets, as a measure of the platelet response. (**c**) Platelet function in coagulation reflected by the ROTEM maximum clot firmness and (**d**) clot formation time. Dashed lines indicate normal ranges as per the manufacturer’s information, and shapes reflect biological replicates. Results were compared across groups with repeated-measures ANOVA to compare differences within biological replicates across groups, and paired *t*-tests were used for pairwise comparisons to the water control (* *p* < 0.05). Comparisons not shown were not statistically significant.

The impact of the series of MNPs on platelets was further investigated through flow cytometry and ROTEM (Figure 10). CD62P (P-selectin) is stored in platelet alpha-granules and mobilized to the platelet surface upon platelet activation and subsequent degranulation. Flow cytometry was used for the detection of the surface expression of CD62P (P-selectin) on unstimulated platelets (Figure 10a) and platelets stimulated with 10 μM of ADP (Figure 10b) to measure the baseline activation and the response to an agonist, respectively. Results of the baseline activation illustrated a trend toward increased platelet activation in all MNP-treated conditions, with statistically significant but small effect sizes observed in bare, A, and E MNPs. While not statistically significant due to the small sample size tested here, baseline platelet activation increases with large effect sizes were observed for particle C and, most prominently, particle D. This trend suggests increased platelet activation with coatings consisting of lower concentrations of CD in the presence of MPC. It is unclear why these formulations induce platelet activation, but it may be attributed to the inhomogeneous surface composition indicated by the PDI. Results of baseline platelet activation were largely mirrored in the platelets stimulated with ADP, demonstrating lower levels of additional degranulation in the conditions that had considerable baseline degranulation. If platelets were degranulated in response to the MNPs, it tracks that they would have a reduced additional response to agonists and may show functional deficits in clot formation. 

Next, we analyzed the platelet function in whole-blood coagulation using ROTEM, with platelet-mediated outcomes of the maximum clot firmness and clot formation time. The EXTEM ROTEM reagent was used to probe the function of the extrinsically activated coagulation pathway. Increases in platelet activation with particle C did translate to a statistically significant increase in clot formation time, indicating reduced platelet function, but this was limited to a trend with particle D. No observable trends across replicates were present for the maximum clot firmness. All platelet-dependent ROTEM results remained within the normal ranges, as per the manufacturer’s instructions.

Interestingly, the biological replicate that showed the largest reduction in the platelet count (illustrated in graphs with squares) also showed the most pronounced changes in the platelet function assays, with the largest increase in the unstimulated CD62P surface expression, largest decrease in the maximum clot firmness, and largest increase in the clot formation time for MNP particle D. While the other replicates did not demonstrate effects of the same magnitude, due to the limited sample size tested in these experiments, there is reason to believe that particle D had adverse effects on platelets. This was further supported by additional experiments tested at higher MNP concentrations (0.5 mg/mL), where particle D’s adverse effects were exaggerated with much larger effect sizes, reaching a statistically significant decrease in the maximum clot firmness (Figure S2).

## 4. Conclusions

We assessed the biocompatibility of novel p(PMβCD-co-MPC) magnetic nanoparticles through various techniques, including circular dichroism, fluorescence spectrometry, plasma calcification, the BCA assay, and immunoblot. Circular dichroism showed that bare MNPs caused the highest decrease (3.5%) in the helix structure of α-lactalbumin, while other modified particles caused less than a 1.7% decrease. Particle D caused an 8.4% reduction in the helix structure and a 5.7% increase in the random coil structure. No significant structural alterations were observed in lysozyme. Fluorescence experiments showed that all particles quenched the tryptophan of HSA as the concentration increased. Particle C had the weakest binding affinity towards HSA, whereas bare MNPs exhibited the strongest binding affinity. Particle E had the lowest number of binding sites, whereas particle D had the highest. The zeta potential results showed that all coatings had an effect on the surface charge. However, surfaces B, C, and E were statistically similar, and even though D was statistically more negative, the difference was less than 10 mV.

We observed an obvious resuming effect in the plasma clotting experiment, mirrored by a fibrinogen-dependent outcome in whole-blood coagulation tests. The incubation of modified particles with uremic plasma resumed the turbidity and prolonged the clotting formation time point, among which particles B, C, and D represented the best results. The BCA assay indicated that the modified particles had less protein adsorption compared to the bare MNP. Furthermore, we assessed the adsorption of 21 plasma proteins to MNPs through immunoblot analysis. It was found that only 12 proteins were adsorbed to MNPs. The lack of proteins S and C in the samples could imply a limited fibrinolytic response. Kininogen, which participates in initiating the contact activation pathway, was not found. The absence of kininogen in the protein corona of MNPs might provide insight into more suitable surfaces for blood-contacting materials. It is even more pertinent to note that kininogen (high molecular weight) can be adsorbed to hemodialysis membranes, where surface modification and ionic strength play an essential role in tuning the surface [82,83]. In terms of the whole-blood hemocompatibility tested on *n* = 3 biological replicates, no robust MNP-dependent effects were observed on the erythrocyte or leukocyte counts. Particle D showed a trend toward decreased platelet counts, suggesting increased aggregation, which was mirrored in the flow cytometry results, suggesting increased platelet activation. Contradictory results from the previous literature make it difficult to ascertain if a small change in the surface charge accounts for increased platelet activity [84,85]. It is likely mediated by the bound proteins more than the underlying surface charge. Particle C caused a statistically significant increase in hemolysis, though the results remained within acceptable levels of hemolysis and slight activation of platelets, which resulted in a statistically significant increase in the clot formation time. Overall, this work provided a comprehensive evaluation of the hemocompatibility of p(PMβCD-co-MPC)-engineered surfaces, reinforcing this polymer combination’s potential for use in the context of chronic kidney disease.

## Figures and Tables

**Table 1:** The representative synthesis scheme for A to E particle series.

	A	B	C	D	E
PMβCD:MPC	4:0	3:1	2:2	1:3	0:4
PMβCD (mmol)	1.25	0.9375	0.625	0.3125	0
MPC (mmol)	0	0.3125	0.625	0.9375	1.25

**Table 2:** Representative results for the effects of particles A to E and MNP on the secondary structure of α-lactalbumin, human serum albumin, and lysozyme, as determined using CD spectra between 190 and 260 nm. The results are an average of three repeated scans. The unit is in percentage (%). Data represent mean ± 1 SD, *n* = 3.

	Particle	Helix	Beta-Sheet	Beta-Turn	Random Coil
Lysozyme	Blank	33.8 ± 0.1	19.7 ± 0.2	18.1 ± 0.0	28.4 ± 0.3
	A (1:0)	30.4 ± 0.2	22.1 ± 0.1	18.0 ± 0.1	29.6 ± 0.1
	B (3:1)	30.7 ± 0.1	21.8 ± 0.3	18.0 ± 0.0	29.5 ± 0.0
	C (1:1)	31.1 ± 0.1	21.3 ± 0.2	18.0 ± 0.0	29.6 ± 0.3
	D (1:3)	30.6 ± 0.3	21.9 ± 0.2	18.0 ± 0.0	29.9 ± 0.5
	E (0:4)	31.4 ± 0.1	21.0 ± 0.1	17.9 ± 0.1	29.7 ± 0.2
	Bare MNP	30.7 ± 0.2	21.8 ± 0.3	17.9 ± 0.1	29.5 ± 0.1
Human serum albumin	Blank	71.0 ± 0.1	4.1 ± 0.2	12.6 ± 0.0	12.3 ± 0.2
	A (1:0)	71.0 ± 0.2	4.1 ± 0.1	12.6 ± 0.1	12.3 ± 0.2
	B (3:1)	71.1 ± 0.3	4.1 ± 0.4	12.6 ± 0.0	12.3 ± 0.2
	C (1:1)	67.4 ± 0.1	5.4 ± 0.2	12.9 ± 0.2	14.3 ± 0.3
	D (1:3)	62.6 ± 0.5	6.0 ± 0.1	13.4 ± 0.3	18.0 ± 0.2
	E (0:4)	68.7 ± 0.2	6.5 ± 0.2	13.0 ± 0.2	11.8 ± 0.1
	Bare MNP	70.3 ± 0.1	4.2 ± 0.3	12.7 ± 0.1	12.7 ± 0.1
α-Lactalbumin	Blank	31.7 ± 0.2	22.1 ± 0.3	18.2 ± 0.0	28.0 ± 0.3
	A (1:0)	31.7 ± 0.1	22.1 ± 0.2	18.2 ± 0.1	28.0 ± 0.2
	B (3:1)	30.8 ± 0.3	22.4 ± 0.2	18.2 ± 0.1	28.6 ± 0.1
	C (1:1)	31.4 ± 0.4	21.7 ± 0.2	18.1 ± 0.1	28.7 ± 0.2
	D (1:3)	30.0 ± 0.1	22.9 ± 0.3	18.1 ± 0.0	29.0 ± 0.3
	E (0:4)	31.3 ± 0.2	22.1 ± 0.4	18.2 ± 0.1	28.5 ± 0.2
	Bare MNP	28.2 ± 0.2	22.4 ± 0.3	21.1 ± 0.1	28.4 ± 0.1

**Table 3:** Summary of platelet-poor plasma clotting initiation and completion time of the non-uremic toxin group, 1 h incubation group, and 4 h incubation group (*n* = 3, all SDs are less than 3 s).

	A	B	C	D	E	Bare MNP	Plasma
Turbidity	0.67	0.57	0.64	0.45	0.74	0.79	0.93
Clotting starting point (min)	2	1	1	2	2	2	10
Plateau point (min)	5	5	5	6	6	6	30

**Table 4:** Relative intensities of immunoblot of plasma proteins adsorbed to different types of MNPs.

Plasma Protein	Fragment Size (kDa)	Bare MNP	A PMβCD:MPC (4:0)	B (3:1)	C (2:2)	D (1:3)	E (0:4)
Fibrinogen	68	7	8	8	7	7	6
	56	6	8	8	7	4	4
	48	6	7	8	6	4	4
	<48	4	3	5	2	0	0
α_1_-Antitrypsin	54	9	8	9	8	5	5
Prothrombin	72	2	2	2	2	1	2
Vitronectin	54	8	8	10	8	5	6
Prekallikrein	85 50	5 10	2 8	5 9	2 7	1 2	2 5
Antithrombin	53	6	6	4	4	3	3
IgG	55	7	4	3	8	5	5
	27	8	7	8	9	5	5
Albumin	66	9	7	7	8	9	9
Plasminogen	91	9	8	7	8	5	3
C3	187	0	0	0	0	0	0
	115	5	4	4	5	3	2
	70	10	10	10	9	7	8
	42	5	8	8	7	3	3
Factor XII	80	3	2	1	2	1	1
Factor XI	70	9	8	9	10	8	8
Transferrin	77	8	8	7	10	10	8

## Data Availability

The data used to support the findings of this study are included in the article and Supplementary Materials.

## Supplementary Materials

The following supporting information can be downloaded at: https://www.mdpi.com/article/10.3390/biom13081165/s1, Figure S1: Representative reassembled western blot membrane of eluted plasma proteins (A: bare MNPs, B: Particle A, C: Particle E). The middle strips (1-21) are processed in western blots, and two side pieces are non-specifically stained with colloidal gold stain; Figure S2: Increased platelet activation and deficit in platelet function with increased concentration of MNP-D; Table S1: Primary antibodies against human plasma proteins used in immunoblot studies.
