# Supplementary material for: Hemocompatibility of β-Cyclodextrin-Modified (Methacryloyloxy)ethyl Phosphorylcholine Coated Magnetic Nanoparticles
Source: Biomolecules. 2023 Jul 25;13(8):1165. doi: 10.3390/biom13081165 (PMC10452919; doi:10.3390/biom13081165)
Supplement: Supplementary file 1 [file biomolecules-13-01165-s001.zip › biomolecules-2403567-supplementary.pdf]

biomolecules

MDPI

Support information for

Hemocompatibility of β-Cyclodextrin-Modified (Methacryloyloxy)ethyl Phosphorylcholine Coated Magnetic Nanoparticles

Shuhui Li [1,†], Mehdi Ghaffari Sharaf [1,†], Elyn M. Rowe [2], Katherine Serrano [2], Dana V. Devine [2] and Larry D. Unsworth [1,*]

[1] Department of Chemical and Materials Engineering, University of Alberta, Edmonton, AB T6G 1H9, Canada; shuhui1@ualberta.ca (S.L.); mghaffar@ualberta.ca (M.G.S.)

[2] Department of Pathology and Laboratory Medicine, University of British Columbia, Vancouver, BC V6T 1Z7, Canada; elyn.rowe@ubc.ca (E.M.R.); katherine.serrano@blood.ca (K.S.); ddevine@pathology.ubc.ca (D.V.D.)

* Correspondence: lunswort@ualberta.ca

† These authors contributed equally to this work.

Keywords: polymer coated magnetic nanoparticles; cyclodextrin; protein adsorption

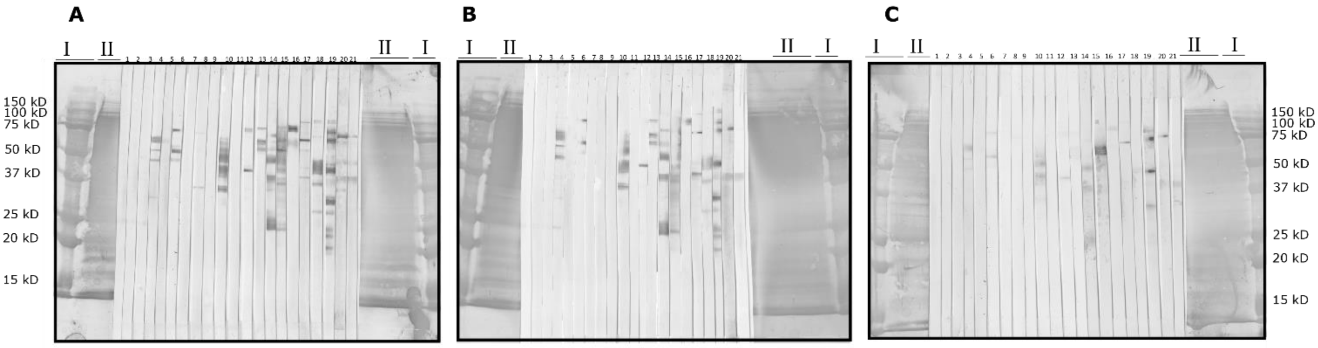

Figure S1. Representative reassembled western blot membrane of eluted plasma proteins (**A**) bare MNPs, (**B**) Particle A, (**C**): Particle E). The middle strips (1-21) are processed in western blots, and two side pieces are non-specifically stained with colloidal gold stain. .

I	Ladder
II	Protein sample stained with non-specific colloidal gold
1	Ani-kininogen (light)
2	Ani-kininogen (heavy)
3	Anti-factor I
4	Anti-fibrinogen α, β, and γ
5	Anti-fibronectin
6	Anti-Alpha$_1$ antitrypsin
7	Anti-thrombin
8	Anti-prothrombin
9	Anti-protein C

10	Anti-vitronectin
11	Anti-protein S
12	Anti-Prekallikrein
13	Anti-antithrombin
14	Anti-IgG
15	Anti-human albumin
16	Anti-plasminogen
17	Anti-C3
18	Anti-factor XII
19	Anti-factor XI
20	Anti-transferrin
21	Anti-alpha$_2$ macroglobulin

Table S1. Primary antibodies against human plasma proteins used in immunoblot studies.

Anti-Human Plasma Antibody	Host	Vendor
Albumin	Goat	OEM Concepts, Saco, ME, USA
Antithrombin	Sheep	Cedarlane Laboratories, Hornby, Ontario, Canada
Complement factor 3	Goat	Calbiochem, Gibbstown, NJ, USA
Factor I	Mouse	Invitrogen; Thermo Fisher Scientific Inc.
Factor XI	Goat	Cedarlane Laboratories, Hornby, Ontario, Canada
Factor XII	Goat	Cedarlane Laboratories, Hornby, Ontario, Canada
Fibrinogen	Rabbit	Calbiochem, Gibbstown, NJ, USA
Fibronectin	Rabbit	Cedarlane Laboratories, Hornby, Ontario, Canada
IgG	Goat	Sigma-Aldrich, St. Louis, MO, USA
Kininogen (heavy chain)	Mouse	US Biological, Swampscott, MA, USA
Kininogen (light chain)	Mouse	US Biological, Swampscott, MA, USA
Plasminogen	Goat	Cedarlane Laboratories, Hornby, Ontario, Canada
Prekallikrein	Sheep	Cedarlane Laboratories, Hornby, Ontario, Canada
Protein C	Sheep	Cedarlane Laboratories, Hornby, Ontario, Canada
Protein S	Sheep	Cedarlane Laboratories, Hornby, Ontario, Canada
Prothrombin	Sheep	Cedarlane Laboratories, Hornby, Ontario, Canada
Thrombin	Sheep	Cedarlane Laboratories, Hornby, Ontario, Canada
Transferrin	Goat	Sigma-Aldrich, St. Louis, MO, USA
Vitronectin	Sheep	Cedarlane Laboratories, Hornby, Ontario, Canada
α1-Antitrypsin	Sheep	Cedarlane Laboratories, Hornby, Ontario, Canada
α2-Macroglobulin	Goat	Sigma-Aldrich, St. Louis, MO, USA

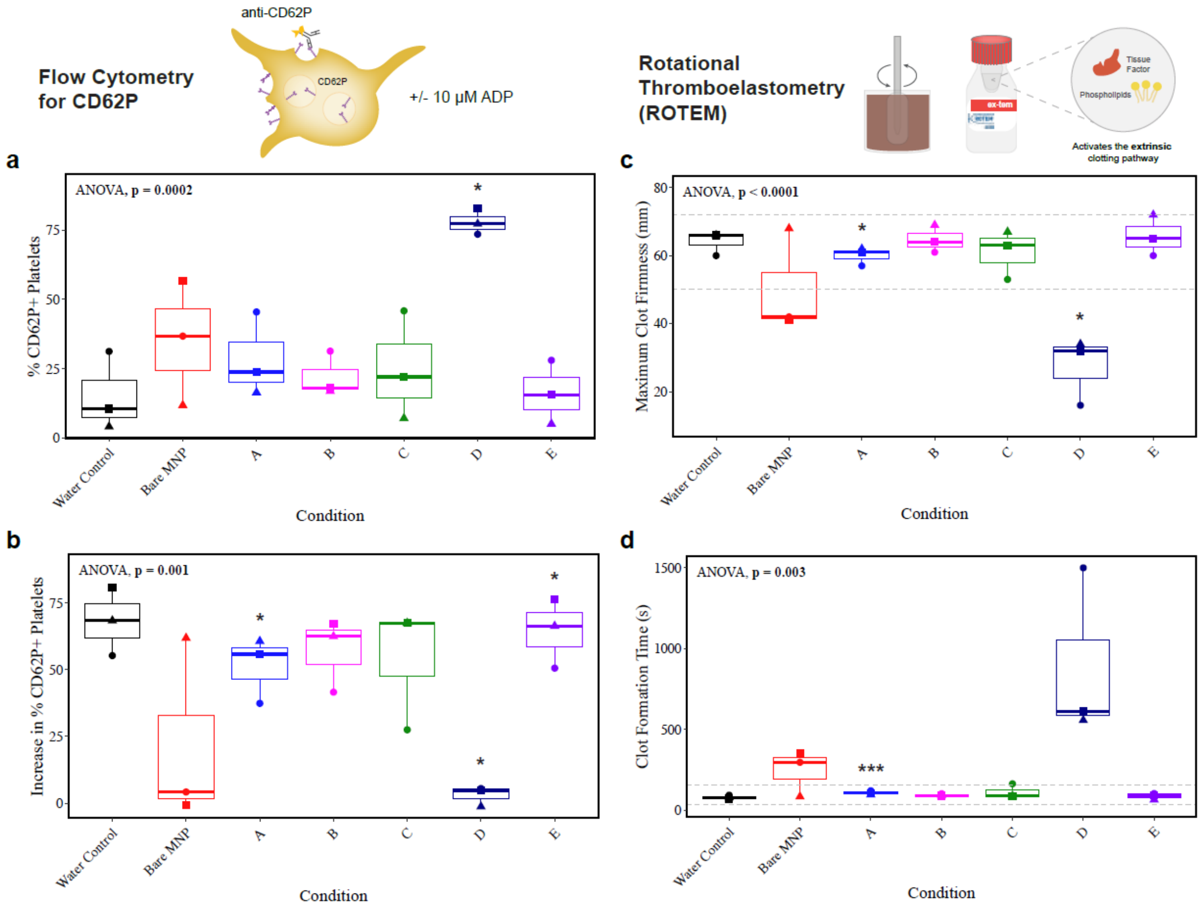

Figure S2. Increased platelet activation and deficit in platelet function with increased concentration of MNP-D. Whole blood from n=3 healthy donors was incubated with 0.5mg/mL of each MNP formulation, then assessed for platelet-related outcomes. (**a**) Baseline platelet activation reflected by surface expression of CD62P detected by flow cytometry. Percentage of CD62P+ platelets displayed. (**b**) Platelet degranulation in response to 10uM ADP, reflected by surface expression of CD62P detected by flow cytometry. Baseline activation was subtracted from the % CD62P+ platelets to yield the increase in degranulated platelets, as a measure of the platelet response. c) Platelet function in coagulation reflected by ROTEM maximum clot firmness and d) clot formation time. Dashed lines indicate normal ranges as per manufacturer's information, and shapes reflect biological replicates. Results were compared across groups with repeated measures ANOVA to compare differences within biological replicates, across groups, and paired t-tests were used for pairwise comparisons to the water control (* $p < 0.05$, *** $p < 0.001$). Comparisons not shown were not statistically significant.
